# Therapeutic Effect of *Ficus lacor* Aerial Roots of Various Fractions on Adjuvant-Induced Arthritic Rats

**DOI:** 10.1155/2013/634106

**Published:** 2013-09-17

**Authors:** Rakesh K. Sindhu, Sandeep Arora

**Affiliations:** Chitkara College of Pharmacy, Chitkara University, Chandigarh-Patiala NH-64, Rajpura, Patiala 140401, Punjab, India

## Abstract

The present study was carried out to evaluate antiarthritic potential and phytochemical screening of various extracts of *Ficus lacor* aerial roots. The antiarthritic activity was evaluated by adjuvant-induced arthritis at the dose of 50 and 100 mg/kg body weight and the standard drug used was indomethacin. The extracts administered in higher doses reduced the lesions to a greater extent showing a dose-dependent decrease in lesions comparable with standard drug indomethacin. The extracts of FLPE and FLET showed significant increase in body weight as compared to arthritic control group as well as an increase in liver weight, a decrease in liver weight, and an increase in spleen weight in arthritis control. The extracts of FLPE and FLET showed significant decrease in WBC count, increase in hemoglobin contents, and RBC count as compared to control group. FLEA and FLCF were not able to produce a significant effect. There was significant reduction in production of IL-1 and TNF-**α** level between model group and control group in serum. In conclusion, we demonstrate that, at 100 mg/kg body weight, doses of FLPE and PLET extracts were highly effective in preventing and suppressing the development of adjuvant-induced arthritis.

## 1. Introduction

Inflammation is common and essential protective response to the harmful stimuli such as infectious agents, antigen-antibody reactions, thermal, chemical, and physical agents, and ischemia [1]. It is caused by a variety of stimuli including physical damage, ultraviolet irradiation, microbial invasion, and immune reactions. The classical key features of inflammation are redness, warmth, swelling, and pain. Inflammation cascades can lead to the development of diseases such as arthritis, chronic asthma, multiple sclerosis, inflammatory bowel disease, and psoriasis. Many of these diseases are debilitating and are becoming increasingly common in our aging society. Rheumatoid arthritis and osteoarthritis are the major inflammatory diseases affecting people worldwide [2]. Rheumatoid arthritis is an inflammatory condition that usually affects multiple joints. It affects 0.3%–1.0% of the general population and is more prevalent among women in the developed countries. Persistent inflammation leads to joint destruction, but the disease can be controlled with drugs. Osteoarthritis, which is characterized by loss of joint cartilage that leads to pain and loss of function primarily in the knees and hips, affects 9.6% of men and 18% of women aged more than 60 years. Increases in life expectancy and aging populations are expected to make osteoarthritis the fourth leading cause of disability by the year 2020 [3]. The pathological changes, causes, mediators, and threat are observed in acute and chronic inflammations. Inflammations are generally characterized by certain regular events such as redness, swelling, heat, pain and at certain times lead to exudation and loss of function. The process of inflammation involves several events and mediators which are potent chemical substances found in the body tissues, such as prostaglandins, leukotrienes, prostacyclins, lymphokines and chemokines like interferon-*α* (IFN-*α*), *γ*, interleukin-(IL-) 1, IL-8, histamine, 5-hydroxytryptamine (5-HT), and tissue necrosis factor-*α* (TNF-*α*) [4]. Anti-inflammatory drugs of synthetic origin are classified as steroidal and nonsteroidal anti-inflammatory agents. The origin of these chemical compounds started when salicylates were isolated from leaf extract of willow bark *Salix alba* and were potentially used by the people of North America in 200 BC and regarded as first generation anti-inflammatory agents [5]. The second- and third-generation compounds with preferential and selective cyclooxygenase (COX2) inhibitory activities, like nimesulide, nabumetone, celocoxib, rofecoxib, valdecoxib, etoricoxib, were discovered. Apart from the nonsteroidal drugs, various corticosteroids such as hydrocortisone, betamethasone, and beclomethasone are primarily used as anti-inflammatory agents [6]. 

*Ficus lacor* Buch.-Ham is synonym of *Ficus infectoria* Roxb. It is locally known as pilkhan and it is a large deciduous, rapidly growing closely foliaceous tree near about 20 meter height with fine shaped crown. It is widely distributed in tropical and subtropical regions of the world. It also grows in various humid regions in India [7]. The bark of the plant in traditional system of India is used for treatment of ulcers, for expelling round worms, and for treatment of leucorrhoea. The leaves are also used for treatment of various skin problems [8, 9]. *Ficus lacor* leaves have been reported the presence of several compounds including *α*-amyrin, *β*-amyrin, lupeol, stigmasterol, and compesterol. The other compounds such as infectorin, scutellarein, scutellarein glucoside, sorbifolin, and bergapten, bergaptol were isolated from the whole plant [10, 11]. The present study was carried out to evaluate anti-arthritic potential of various fractions of *Ficus lacor* aerial roots fractions and phytochemical screening also performed. 

## 2. Materials and Methods

### 2.1. Plant Material

The plant of *Ficus lacor* aerial roots were collected during the month of July 2009 from Panchkula Sector-17 (Haryana), North India. The plant material was taxonomically identified and authenticated by Dr. H. B. Singh, Head of Raw materials Herbarium and Museum Division, with reference no. NISCAIR/RHMD/Consult/2010-11/1638/236. The voucher specimen has been deposited in the herbarium section of the Phytochemistry and Pharmacognosy Division, Chitkara College of Pharmacy, Chitkara University, Punjab, for further reference. The root was dried under shade, sliced into small pieces, pulverised using a mechanical grinder, and stored in an air tight container for further use. 

### 2.2. Preparation of the Extract

About 1 kg of air dried powdered root was extracted with ethanol in a soxhlet extractor for 72 h. The aqueous extract was prepared by maceration with distilled water for 24 h. Concentrated ethanol and aqueous extract in rotary vaccum evaporator and crude ethanol extract were fractioned, namely, petroleum ether (FLPE), ethyl acetate (FLEA), chloroform (FLCF), and ethanol (FLET). 

## 3. Phytochemical Screening [12]

### 3.1. Test for Alkaloids

Stirr a small portion of the solvent-free petroleum ether, chloroform, ethyl acetate, alcohol and water extracts separately with a few drops of dilute hydrochloric acid and filter. The filtrates were tested with various alkaloidal reagents such as Mayer's reagent (cream precipitate), Dragendorff's reagent (orange brown precipitate), and Wagner reagent (reddish brown precipitate). In Mayer's reagent, few drops of mayer's reagent were added in each extract and formation of the white or cream colored precipitates were observed.

*Dragendorff's Reagent.* Few drops of dragendorff's reagent were added in each extract and formation of the orange yellow or brown colored precipitates was observed.

*Wagner Reagent.* Few drops of wagner reagent were added in each extract and observed formation of the reddish brown precipitates. 

### 3.2. Test for Carbohydrates

Dissolve small quantities of alcoholic and aqueous extracts separately in 4 mL of distilled water and filter. The filtrate may be subjected to various tests to detect the presence of carbohydrates.

*Molisch's Test.* About 2 ml of extract and few drops of *α*-naphthol (20% in ethyl alcohol) were added. Then about 1 mL of concentrated sulphuric acid was added along the side of the tube. Reddish violet ring appeared at the junction of two layers which indicates the presence of carbohydrates.

*Fehlings Test.* About 1 mL of Fehling's reagent (copper sulphate in alkaline conditions) was added to the filtrate of the root extract in distilled water and heated in a steam bath. Brick red precipitates appeared which confirms the presence of carbohydrates. 

### 3.3. Test for Glycosides

Another small portion of the extract was hydrolysed with diluted hydrochloric acid for few hours in water bath and subjected the hydrolysate with Liebermann-Burchard's, Keller-Killani, and borntrager's tests to detect the presence of different glycosides. 

*Keller-Killani Test*. 1 mL of glacial acetic acid containing traces of FeCl_3_ and 1 mL of concentrated H_2_SO_4_ was added to the extract carefully. Colour appeared which confirm the presence of glycosides in the root extracts.

*Borntrager's Test.* About 1 mL of benzene and 0.5 mL of dilute ammonia solution were added to the extract. A black brown colour was obtained which shows the presence of glycosides in the root extracts. 

### 3.4. Test for Phenolic Compound and Tannins

Take small quantities of alcohol and aqueous extracts separately in water and test for the presence of phenolic compounds and tannins with dilute ferric chloride solution (5%) and lead acetate test.

*Ferric Chloride Test*. On addition of ferric chloride solution (5%), colour was observed in all the three portions due to the presence of phenolic compounds. 

*Lead Acetate Test*. Few drops of lead acetate solution (5%) were added to the alcoholic extract of the root. White precipitate appeared which confirms the presence of phenolic compounds. 

### 3.5. Test for Flavonoids

*Ammonia Test.* Filter paper strips were dipped in the alcoholic and aqueous solutions of the extract and ammoniated. The filter paper changed its colour to yellow which indicates the presence of flavonoids.

*Pew Test for Flavonoids.* To 1 ml of the each extracts, a piece of metallic magnesium/zinc was added followed by addition of 2 drops of concentrated hydrochloric acid. A brownish colour confirmed the presence of flavonoids in all the extract. 

### 3.6. Test for Proteins and Free Amino Acids

Few milliliters of alcoholic and aqueous extracts were added to a few milliliters of distilled water and subjected to Million's, Biuret and Ninhydrin's tests. 

*Millon's Test.* To 2 mL of filtrate, 5-6 drops of Million's reagent (solution of mercury nitrate and nitrous acid) were added. A red colour precipitate appeared which confirms the presence of proteins and free amino acids.

*Biuret Test*. To the ammoniated alkaline filtrate 2-3 drops of 0.02% copper sulphate solution was added. A red colour was obtained which confirms the presence of proteins and free amino acids. 

*Ninhydrin Test.* To each of the filtrates, lead acetate solution was added to precipitate tannins and filtered. The filtrate was spotted on a paper chromatogram, sprayed with ninhydrin reagent, and dried at 110°C for 5 minutes. Violet spots were seen which confirms the presence of proteins and free amino acids. 

### 3.7. Test for Saponin

*Foam Test*. Dilute 1 mL of alcoholic and aqueous extracts separately with distilled water to 20 mL and shake in a graduated cylinder for 15 minutes. A one centimeter layer of foam indicates the presence of saponin.

*Sodium Bicarbonate Test.* To the few milligrams of extract, few drops of sodium bicarbonate were added and shaken well. Formation of honey comb like frothing indicates positive test for saponins. 

### 3.8. Test for Phytosterol and Triterpenes

*Liebermann-Burchard's Test.* The hydroalcoholic extract was shaken with chloroform and few drops of acetic anhydride were added to chloroform extract along with a few drops of concentrated sulphuric acid from the side of the tube. The appearance of blue to brick red colour indicates the presence of sterol and triterpenes.

*Hesse's Reaction.* The residue was dissolved in chloroform (4 mL) and an equal quantity of concentrated sulphuric acid was then along the side of the tube. The formation of the pink colored ring, which results from shaking, diffused in both layers, indicating the presence of sterols in the extract.

#### 3.8.1. TLC for Alkaloids

 Presence of alkaloids in various extracts was investigated by using the Ethyl acetate : methanol : water (100 : 13.5 : 10) mobile phase. The development was done by using 0.5% anisaldehyde reagent as the developing agent and observation of spots was done either in normal or UV light. Following observation was made [13]. 

#### 3.8.2. TLC for Glycosides

 Presence of glycosides in various extracts was investigated by using toluene: ethyl acetate (70 : 30) mobile phase. The development was done by using 0.5% Anisaldehyde reagent as the developing agent and observation of spots was done either in normal or UV light [13]. 

#### 3.8.3. TLC for Flavonoids

 Presence of flavonoids in various Extracts was investigated by using the ethyl acetate : formic acid : glacial acetic acid : water (100 : 11 : 11 : 26) mobile phase. The development was done by using 0.5% anisaldehyde reagent as the developing agent and observation of spots was done either in normal or UV light [13]. 

### 3.9. Animals

The Wistar rats (160–220 g) were acclimatized to the standard laboratory conditions (temperature 25 ± 2°C) and maintained on 12 hr light, 12 hr dark cycle. The animals were fed with standard diet and water *ad libitum*. The animals were maintained as per the norms of CPCSEA and the experiments were cleared by CPCSEA and the institutional ethics committee. The experimental protocol was approved by the Institutional Animal Ethical Committee (IAEC) constituted under CPCESA (Chitkara College of Pharmacy Animal Facility registration number: 1181/ab/08/CPCSEA and protocol approval number: IAEC/CCP/12/PR-005).

## 4. Toxicity Studies

Acute toxicity study was performed for ethanol extract according to the acute toxic classic method as per OECD guidelines [14]. Albino rats were used for acute toxicity study. The animals were kept fasting for overnight providing only water, after which the extract was administered orally at the dose of 100 mg/kg and observed for 14 days. If mortality was observed in two animals out of three animals, then the dose administered was assigned as toxic dose. If the mortality was observed in one animal, then the same dose was repeated to confirm the toxic dose. If mortality was not observed, the procedure was repeated for further higher doses such 50, 200 and 2000 mg/kg body weight. The animals were observed for toxic symptoms such as behavioral changes, locomotion, convulsions and mortality for 72 hr.

## 5. Adjuvant Induced Arthritis [15]

Animals were divided into 9 groups and each group 6 animals. Arthritis was induced by intradermal injection of 0.05 mL of a 5 mg/mL suspension of heat killed *Mycobacterium tuberculosis* in liquid paraffin into the plantar surface of the hind paws. The clinical features of adjuvant induced arthritis (AIA) manifested as erythema, induration and edema and presented in multiple joints as follows: (a) onset: clinical signs around days 8–10; (b) early phase: progressive severity of the clinical signs over the next 7–10 days; (c) late phase: spontaneous regression during the next 10–14 days.

All four paws were examined and graded for severity and loci of the arthritic lesions (erythema, swelling, and induration) which developed on the paws by the 15th day and assessed on an arbitrary 5-point scale of arthritic score (0–4). Rats were assessed daily for signs of arthritis up to 28th day after-CFA. The maximal arthritic score per rat was set at 16 (maximum of 4 points × 4 paws), where

 0 = no signs of disease;
 1 = signs involving the ankle/wrist;
 2 = signs involving the ankle plus tarsals (proximal part of the hind paw) and/or wrist plus carpals of the forepaw;
 3 = signs extending to the metatarsals or metacarpals; and
 4 = severe signs involving the entire hind or fore paw.

Animals with pronounced arthritis were separated into groups of 6, with similar mean arthritic scores and injected with ethanolic extracts and different doses of solvent fractions or reference drug (indomethacin 2.5 mg/kg) intraperitonealy in two doses (one dose corresponding to ED50 dose for fractions as determined for inhibition of carrageenan-induced inflammation, (or 20 mg/kg) and a higher dose well below the toxic dose, which was 100–150 mg/kg for various fractions) daily for next 7 days from the 15th day onwards. Mean arthritic scores obtained for each day after 15th day for treated groups were compared with appropriate scores of the control arthritic group. 

 Considering the changes in body weight on the 7th, 14th, and 21st day and at the end of the 28th day, rats were housed in the metabolic cages and their urine collected for 24 hours in beakers maintained at 0°C in ice bath. Rats were sacrificed on the 29th day by decapitating. The blood was collected and biochemical parameters like haemoglobin content, total RBC counts, WBC counts and Erythrocyte Sedimentation Rate (ESR) were estimated. Plasma was separated from the blood collected with EDTA and IL-1 and TNF-*α* were measured. Immediately after sacrificing, liver, kidney, and spleen were separated, regarding organ weight changes on the 28th day. 

## 6. Statistical Analysis

Data obtained from animal experiments were expressed as mean standard error (±S.E.M.). Statistical differences between the treatments and the control were evaluated by ANOVA and Student-Newman-Keuls post hoc tests. Significance of data was expressed as *P < 0.05, **P < 0.01, and ***P < 0.001.

## 7. Results 

**Figure 1:**
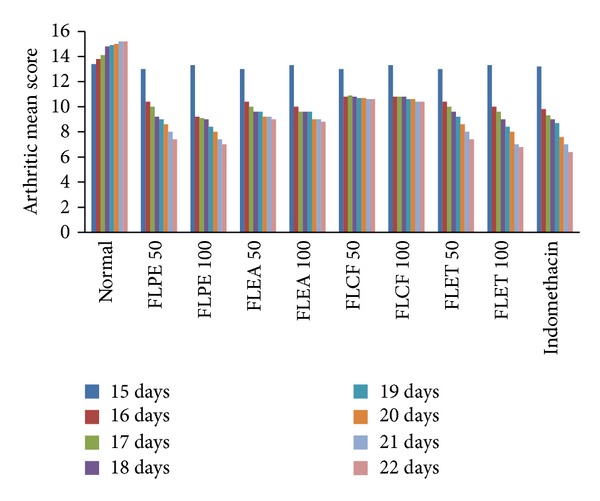
Inhibition of arthritic score by various extracts.

**Figure 2:**
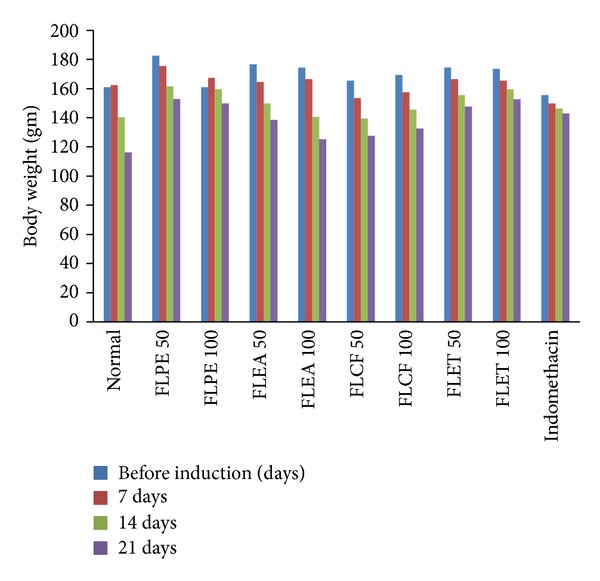
Changes in body weight of adjuvant induced arthritis.

**Figure 3:**
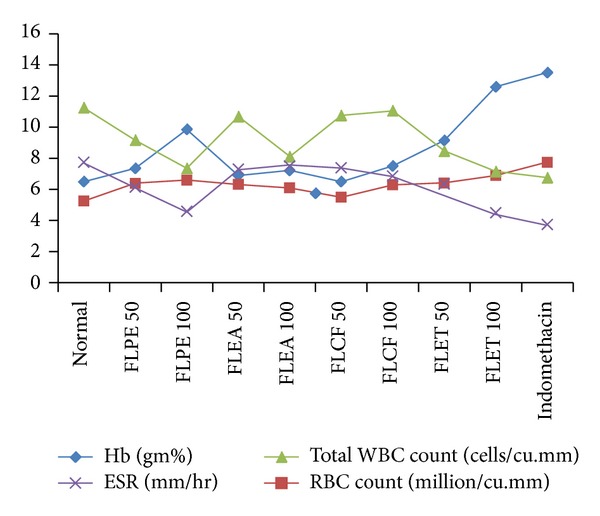
Haematological parameters in adjuvant induced arthritis.

The phytochemical screening of different extracts shows the presence of Glycosides, alkaloids, phenolic and tannins, sterol and Flavonoids. The TLC of alkaloids shows different spots having Rf value, that is, 0.45, 0.58, and 0.62. The TLC of glycosides shows different spots having Rf values, that is, 0.42 and 50. The TLC of flavonoids shows different spots having Rf values, that is, 0.54, 0.61, 0.64 and 68. FLPE and FLET (Table 1 and Figure 1) showed statistically significant inhibition of arthritic lesions (*P* < 0.05) from day 16, (*P* < 0.01) from day, 20 and (*P* < 0.001) from day 21 onwards. Showing the extracts administered in higher doses reduced the lesions to a greater extent a dose-dependent decrease in lesions comparable with standard drug indomethacin. The extracts FLPE and FLET showed significant increase in body weight (*P* < 0.001) as compared to arthritic control group and increase in liver weight (*P* < 0.01), decrease in liver weight (*P* < 0.001), and increase in spleen weight (*P* < 0.001) in arthritis control (Tables 2 and 3 and Figure 2). FLEA did not show any significant result in body and organ weight estimation. The extracts FLPE and FLET showed significant decrease in WBC count (*P* < 0.001) and increase in hemoglobin contents and RBC counts as compared to control group (Table 4 and Figure 3). FLEA and FLCF were not able to produce a significant effect. There was significant difference between model group and control group in IL-1 and TNF-*α* level. After treatment with FLPE, FLEA, FLCF, and FLET the level of IL-1 and TNF-*α* in serum was lower than arthritic control group in FLPE and FLET extracts (Table 5).

## 8. Discussion

The present investigations establishe the antiarthritic potential of FLPE and FLET extracts using adjuvant induced arthritis model rats because rats develop a chronic swelling in multiple joints, with the influence of inflammatory cells, erosion of joint cartilage, and bone destruction. It is very close to human arthritis disease [16]. The chronic inflammation involves the release of various types of mediators like cytokines, interferons, and prostaglandins. These mediators are responsible for the pain and destruction of cartilage and bone that can lead to severe disability [17]. However, the standard drug, indomethacin, and extracts of FLPE and FLET significantly suppressed the inflammation of the rat paws. In arthritic state, there is a mild to moderate rise in WBC count due to the release of IL-I inflammatory response, and IL-I increases the production of both granulocyte and macrophages colony stimulating factors. In the present study, the migration of leucocytes into the swelled area is significantly suppressed by FLPE and FLET extracts when compared to standard drug indomethacin, as seen from the significant reduction in the total WBC count. Erythrocyte sedimentation rate is an estimation of the suspension stability of RBC's in plasma. It is associated to the number and size of the red cells and with the relative concentration of plasma proteins, especially fibrinogen, alpha, and beta globulins. Increase in the rate, is an indication of active but obscure disease processes. The acute phase proteins in ESR and C-reactive proteins (CRP) share the property of showing elevations in the concentration in response to stress or inflammations like injection, injury, surgery, and tissue necrosis. The ESR count significantly increased in arthritic control group, whereas these counts were remarkably counteracted in the standard indomethacin and FLPE and FLET extracts groups and thus justifying its significant role in the arthritic conditions [18]. Changes in body weight have also been used to consider the course of the disease and the response to therapy of anti-inflammatory drugs [19]. As the incidence and severity of arthritis increased, the changes in the body weights of the rats also occurred during the course of the experimental period. The loss of the body weight during arthritic condition was also supported by earlier observations, with on alterations in the metabolic activities of diseased rats [20]. 

## 9. Conclusion

From the results, it may be concluded that the FLPE and FLET extracts of *Ficus lacor *Buch.-Hum. aerial roots at the dose of 100 mg/kg body weight show a significant antiarthritic activity which maybe due to the presence of various types of phytoconstituents such as alkaloids, steroids, flavonoids, phenolic compounds, and glycosides. Several studies indicate that above rementioned phytoconstituents possess significant antiarthritic activity. The study is further extended to identify and characterize the exact active phytoconstituents and which are responsible for the observed significant antiarthritic activity against adjuvant-induced arthritis in rats.

## Figures and Tables

**Table 1:** Inhibition of adjuvant induced poly arthritis in rats (administered daily from days 15–21) by various extracts from *Ficus lacor*.

Groups	Isolated extracts	Dose mg/kg	Mean arthritic score ± S.E.M. for days							
			15	16	17	18	19	20	21	22
I	Normal	1 mL i.p.	13.4 ± 1.3	13.8 ± 1.4	14.1 ± 1.3	14.8 ± 1.4	14.9 ± 1.5	15.0 ± 1.3	15.2 ± 1.4	15.2 ± 1.5
II	FLPE	50 mg i.p.	13.0 ± 1.4	10.4 ± 1.4	10.0 ± 1.1	9.2 ± 1.0*	9.0 ± 1.0*	8.6 ± 0.9*	8.0 ± 0.8*	7.4 ± 0.8^#^
III	FLPE	100 mg i.p.	13.3 ± 1.3	9.2 ± 1.1*	9.1 ± 1.0*	9 ± 1.1*	8.4 ± 0.9	8 ± 0.8^@^	7.4 ± 0.8^#^	7.0 ± 0.9^#^
IV	FLEA	50 mg i.p.	13.0 ± 1.4	10.4 ± 1.4	10.0 ± 1.1	9.6 ± 1.0	9.6 ± 1.0*	9.2 ± 0.9*	9.2 ± 0.8*	9.0 ± 0.8^@^
V	FLEA	100 mg i.p.	13.3 ± 1.3	10 ± 1.1	9.6 ± 1.0	9.6 ± 1.0	9.6 ± 1.0*	9.0 ± 0.9*	9.0 ± 0.8*	8.8 ± 0.8^@^
VI	FLCF	50 mg i.p.	13.0 ± 1.4	10.9 ± 1.4	10.8 ± 1.1	10.9 ± 1.0	10.7 ± 1.0	10.7 ± 0.9	10.6 ± 0.8*	10.6 ± 0.8*
VII	FLCF	100 mg i.p.	13.3 ± 1.3	10.8 ± 1.1	10.8 ± 1.0	10.8 ± 1.1	10.6 ± 0.9	10.6 ± 0.8	10.4 ± 0.8*	10.4 ± 0.9*
VIII	FLET	50 mg i.p.	13.0 ± 1.4	10.4 ± 1.4	10.0 ± 1.1	9.6 ± 1.0	9.2 ± 1.0*	8.6 ± 0.9*	8.0 ± 0.8*	7.4 ± 0.8^@^
IX	FLET	100 mg i.p.	13.3 ± 1.3	10 ± 1.1	9.6 ± 1.0	9 ± 1.1*	8.4 ± 0.9*	8 ± 0.8^@^	7.0 ± 0.8^@^	6.8 ± 0.2^#^
X	Indomethacin	2.5 mg i.p.	13.2 ± 1.2	9.8 ± 1.0*	9.3 ± 1.0*	9 ± 1.2*	8.7 ± 0.8^#^	7.6 ± 0.8^@^	7.0 ± 0.9^@^	6.4 ± 0.8^#^

Group I: arthritic rats treated with saline; Group II: arthritic rats treated with FLPE 50 mg/kg; Group III: arthritic rats treated with FLPE 100 mg/kg; Group IV: arthritic rats treated with FLEA 50 mg/kg; Group V: arthritic rats treated with FLEA 100 mg/kg; Group VI: arthritic rats treated with FLCF 50 mg/kg, Group VII: arthritic rats treated with FLCF-100 mg/kg; Group VIII: Arthritic rats treated with FLET 50 mg/kg, Group IX: arthritic rats treated FLET 100 mg/kg; Group X: arthritic rats treated with indomethacin; *P < 0.05, ^@^ *P* < 0.01, ^#^ *P* < 0.001 as compared to arthritic control.

**Table 2:** Effect of isolated extracts from *Ficus lacor* on changes in body weight in adjuvant induced arthritic rats.

Groups	Isolated extracts	Dose (mg/gm)	Before induction (gm)	After 7 days (gm)	After 14 days (gm)	On 21st day (gm)
I	Normal Arthritic	1 mL	160.85 ± 5.40	162.50 ± 5.20	140.40 ± 6.25	116.35 ± 5.65
II	FLPE	50	182.50 ± 5.33	175.34 ± 5.56	161.45 ± 6.57	152.75 ± 5.90**
III	FLPE	100	180.38 ± 4.68	167.34 ± 5.56	159.45 ± 6.57	149.75 ± 5.70***
IV	FLEA	50	176.52 ± 4.75	164.45 ± 5.37	149.68 ± 5.64	138.45 ± 6.34
V	FLEA	100	174.32 ± 5.35	166.50 ± 4.74	140.58 ± 3.43	125.35 ± 4.39*
VI	FLCF	50	165.45 ± 5.68	153.50 ± 4.79	139.40 ± 6.75	127.50 ± 5.76*
VII	FLCF	100	169.38 ± 3.60	157.45 ± 4.79	145.56 ± 4.75	132.50 ± 5.60*
VIII	FLET	50	174.50 ± 4.86	166.48 ± 5.50	155.50 ± 6.54	147.57 ± 4.48***
IX	FLET	100	173.60 ± 4.65	165.48 ± 5.50	159.50 ± 6.54	152.57 ± 4.48***
X	Indomethacin	2.5	155.44 ± 7.50	149.79 ± 5.75	146.35 ± 5.50	142.90 ± 6.59***

Group I: arthritic control (saline), Group II: arthritic rats treated with from FLPE fraction 50 mg, Group III: arthritic rats treated with FLPE fraction 100 mg, Group IV: arthritic rats treated FLEA fraction 50 mg, Group V: arthritic rats treated with FLEA fraction 100 mg, Group VI: arthritic rats treated with FLCF fraction 50 mg, Group VII: arthritic rats treated with FLCF fraction 100 mg,Group VIII: arthritic rats treated with FLET fraction 50 mg, Group IX: arthritic rats treated with FLET 100 mg from *Ficus lacor,* Group X: arthritic rats treated with indomethacin 2.5 mg/kg. *P < 0.05, **P < 0.01, ***P < 0.001 as compared to arthritic control.

**Table 3:** Effect of isolated extracts from *Ficus lacor* on organ weight changes in adjuvant induced arthritis in rats.

Groups	Extracts	Dose (mg/kg)	Liver (gm)	Thymus (mg)	Spleen (mg)
I	Control arthritic	1 mL	3.65 ± 0.26	87.45 ± 1.58	570.30 ± 2.95
II	FLPE	50	4.25 ± 0.26**	92.60 ± 2.24**	410.35 ± 2.64*
III	FLPE	100	5.39 ± 0.19**	95.48 ± 1.53***	345.58 ± 3.45***
IV	FLEA	50	3.74 ± 0.19	83.50 ± 1.57	425.54 ± 2.45
V	FLEA	100	3.48 ± 0.26	86.55 ± 2.75	530.30 ± 1.54
VI	FLCF	50	3.99 ± 0.34*	90.35 ± 3.70**	558.80 ± 3.47
VII	FLCF	100	4.02 ± 0.31*	90.35 ± 3.70**	489.45 ± 2.89
VIII	FLET	50	4.40 ± 0.20**	95.90 ± 1.50**	399.50 ± 2.89**
IX	FLET	100	5.63 ± 0.13***	102.76 ± 1.54***	338.95 ± 1.75***
X	Indomethacin	2.5	6.68 ± 0.18***	105.40 ± 1.68***	305.40 ± 2.26***

Group I: arthritic control (saline), Group II: arthritic rats treated with from FLPE fraction 50 mg, Group III: arthritic rats treated with FLPE fraction 100 mg, Group IV: arthritic rats treated FLEA fraction 50 mg, Group V: arthritic rats treated with FLEA fraction 100 mg, Group VI: arthritic rats treated with FLCF fraction 50 mg, Group VII: arthritic rats treated with FLCF fraction 100 mg, Group VIII: arthritic rats treated with FLET fraction 50 mg, Group IX: arthritic rats treated with FLET 100 mg from *Ficus lacor,* Group X: arthritic rats treated with indomethacin 2.5 mg/kg. *P < 0.05, **P < 0.01, ***P < 0.001 as compared to arthritic control.

**Table 4:** Effect of isolated extracts from *Ficus lacor* on haematological parameters in adjuvant induced arthritic rats.

Groups	Isolated extracts	Dose (mg/gm)	Hb (gm%)	RBC count (million/cu·mm)	Total WBC count (cells/cu·mm)	ESR (mm/hr)
I	Arthritic control	1 mL	6.50 ± 0.32	5.25 ± 0.10	11.25 ± 0.50	7.75 ± 0.25
II	FLPE	50	7.35 ± 0.15**	6.40 ± 0.35***	9.16 ± 0.40*	6.15 ± 0.18
III	FLPE	100	9.85 ± 0.20***	6.60 ± 0.42***	7.35 ± 0.24**	4.60 ± 0.15**
IV	FLEA	50	6.90 ± 0.14*	6.32 ± 0.45	10.68 ± 0.35	7.30 ± 0.29
V	FLEA	100	7.22 ± 0.19	6.10 ± 0.37	8.10 ± 0.42	7.50 ± 0.25*
VI	FLCF	50	6.50 ± 0.12	5.50 ± 0.33	10.75 ± 0.15	7.38 ± 0.30
VII	FLCF	100	7.50 ± 0.32	6.30 ± 0.30	11.05 ± 0.15*	6.85 ± 0.43*
VIII	FLET	50	9.15 ± 0.30***	6.42 ± 0.28***	8.46 ± 0.28	6.34 ± 0.40**
IX	FLET	100	12.59 ± 0.26***	6.90 ± 0.27***	7.15 ± 0.28***	4.50 ± 0.40***
X	Indomethacin	2.5	13.50 ± 0.10***	7.75 ± 0.20***	6.75 ± 0.15***	3.75 ± 0.27***

Group I: arthritic control (saline), Group II: arthritic rats treated with from FLPE fraction 50 mg, Group III: arthritic rats treated with FLPE fraction 100 mg, Group IV: arthritic rats treated FLEA fraction 50 mg, Group V: arthritic rats treated with FLEA fraction 100 mg, Group VI: arthritic rats treated with FLCF fraction 50 mg, Group VII: arthritic rats treated with FLCF fraction 100 mg, Group VIII: arthritic rats treated with FLET fraction 50 mg, Group IX: arthritic rats treated with FLET 100 mg from *Ficus lacor,* Group X: arthritic rats treated with indomethacin 2.5 mg/kg. *P < 0.05, **P < 0.01, ***P < 0.001 as compared to arthritic control.

**Table 5:** Effect of various extracts from *Ficus lacor* on interleukin-1 (IL-1) and tumor necrosis factor- *α* (TNF-*α*) in serum of adjuvant induced poly arthritis in rats.

Groups	Isolated extracts	Dose (mg/kg)	IL-1	TNF-*α*
I	Normal	1 mL i.p.	0.395 ± 0.084	0.108 ± 0.037
II	FLPE	50	0.575 ± 0.105**	0.178 ± 0.046**
III	FLPE	100	0.510 ± 0.145***	0.159 ± 0.043***
IV	FLEA	50	0.695 ± 0.125	0.193 ± 0.024
V	FLEA	100	0.745 ± 0.165*	0.193 ± 0.035*
VI	FLCF	50	0.720 ± 0.156	0.188 ± 0.029
VII	FLCF	100	0.760 ± 0.176*	0.188 ± 0.044*
VIII	FLET	50	0.595 ± 0.135**	0.170 ± 0.047**
IX	FLET	100	0.495 ± 0.127***	0.155 ± 0.025***
X	Indomethacin	2.5	0.475 ± 0.142***	0.135 ± 0.050***

Group I: arthritic control (saline), Group II: arthritic rats treated with from FLPE fraction 50 mg, Group III: arthritic rats treated with FLPE fraction 100 mg, Group IV: arthritic rats treated FLEA fraction 50 mg, Group V: arthritic rats treated with FLEA fraction 100 mg, Group VI: arthritic rats treated with FLCF fraction 50 mg, Group VII: arthritic rats treated with FLCF fraction 100 mg, Group VIII: arthritic rats treated with FLET fraction 50 mg, Group IX: arthritic rats treated with FLET 100 mg from *Ficus lacor,* Group X: arthritic rats treated with indomethacin 2.5 mg/kg. *P < 0.05, **P < 0.01, ***P < 0.001 as compared to arthritic control.
